# Acacetin from Traditionally Used *Saussurea involucrata* Kar. et Kir. Suppressed Adipogenesis in 3T3-L1 Adipocytes and Attenuated Lipid Accumulation in Obese Mice

**DOI:** 10.3389/fphar.2017.00589

**Published:** 2017-08-29

**Authors:** Chian-Jiun Liou, Shu-Ju Wu, Li-Chen Chen, Kuo-Wei Yeh, Chih-Ying Chen, Wen-Chung Huang

**Affiliations:** ^1^Department of Nursing, Research Center for Chinese Herbal Medicine, Chang Gung University of Science and Technology Taoyuan, Taiwan; ^2^Division of Allergy, Asthma, and Rheumatology, Department of Pediatrics, Chang Gung Memorial Hospital Taoyuan, Taiwan; ^3^Department of Nutrition and Health Sciences, Chang Gung University of Science and Technology Taoyuan, Taiwan; ^4^Aesthetic Medical Center, Department of Dermatology, Chang Gung Memorial Hospital Taoyuan, Taiwan; ^5^Graduate Institute of Health Industry Technology, Research Center for Food and Cosmetic Safety, Research Center for Chinese Herbal Medicine, College of Human Ecology, Chang Gung University of Science and Technology Taoyuan, Taiwan

**Keywords:** 3T3-L1, acacetin, adipogenesis, AMPK, anti-obesity, lipolysis

## Abstract

Acacetin, a flavone that can be isolated from the *Saussurea involucrata* plant, has anti-tumor and anti-inflammatory properties that ameliorate airway hyperresponsiveness in asthmatic mice. This study investigated whether acacetin has anti-adipogenic effects in 3T3-L1 adipocytes and whether it regulates the inflammatory response in adipocytes and macrophages. It also investigated whether acacetin ameliorates lipid accumulation in high-fat diet- (HFD) induced obese mice. Differentiated 3T3-L1 cells were treated with acacetin. The glycerol levels in the culture medium were measured, and the expression of proteins and genes involved in adipogenesis and lipolysis were assayed by Western blot and real-time PCR, respectively. Inflammatory cytokine signaling pathway activity was assessed in macrophages that were treated with acacetin and cultured with differentiated medium from 3T3-L1 cells. Intraperitoneal injections of acacetin were administered to HFD-induced obese mice twice a week for 10 weeks. Acacetin significantly increased the levels of glycerol in the culture medium and significantly inhibited lipid accumulation in adipocytes. Acacetin reduced the expression of adipogenesis-related transcription factors, including the expression of the CCAAT/enhancer-binding protein; it also increased sirtuin 1 expression and AMPK phosphorylation in adipocytes. In macrophages cultured with differentiated media from 3T3-L1 adipocytes, acacetin reduced the levels of inflammatory mediators and the activity of the mitogen-activated protein kinase and NF-κB pathways. In obese mice, acacetin reduced both body weight and visceral adipose tissue weight. These results demonstrate that acacetin inhibited adipogenesis in adipocytes and in obese mice. Acacetin also reduced the inflammatory response of macrophages that were stimulated with differentiated media from 3T3-L1 cells.

## Introduction

Obesity is a global public health problem that is associated with an increasing prevalence of chronic diseases such as metabolic syndrome, type 2 diabetes, cardiovascular disease, and cancer (Smith and Minson, 2012). White adipose tissue is thought to protect organs and to act as an energy store. Excessive triglyceride levels not only cause lipids to accumulate, they also interfere with the physiological function of adipocytes (Sun et al., 2011). Excessive lipid deposits in adipocytes impair insulin sensitivity and lead to metabolic syndrome abnormalities. The sterol regulatory element-binding protein 1c (SREBP-1c), the CCAAT/enhancer-binding protein (C/EBP), and the peroxisome proliferator-activated receptor (PPAR) protein are all important transcription factors that bind to the promoters of lipogenesis genes, which are involved in the synthesis of fatty acids and triglycerides (Romeo et al., 2012). Notably, lipolysis enzymes break down triglycerides to reduce lipid accumulation in adipocytes. Thus, blocking the expression of transcription factors that are important for lipid synthesis will reduce lipid accumulation in adipocytes and reducing the effects of obesity.

A previous study found that in obese individuals, adipose tissue can secrete excessive chemokine ligand 5 (CCL5) and monocyte chemotactic protein-1 (MCP-1) protein to recruit macrophages that infiltrate the adipose tissue (Smith and Minson, 2012). Adipokines and free fatty acids released from adipose tissue stimulate the activity of macrophages, which express the proinflammatory cytokines TNF-α and IL-6; these factors interfere with the physiological function of adipocytes and induce the development of insulin resistance in adipocytes (Klimcakova et al., 2010; Li F. et al., 2017). Several studies have shown that adipokines influence both local and systemic inflammatory processes in obesity (Zagotta et al., 2015; Guerrero-Garcia et al., 2016; Moseti et al., 2016). Inflammatory adipocytes release adipokines or free fatty acids that upregulate the transcriptional regulator nuclear transcription factor kappa-B (NF-κB), which leads to the secretion of IL-6 and TNF-α by macrophages (Sun et al., 2011; Huang et al., 2013). Therefore, reducing macrophage expression of pre-inflammatory cytokines can improve and attenuate metabolic syndrome as well as the development of chronic inflammation and metabolic diseases.

*Saussurea involucrata* Kar. et Kir. mainly grows in the high mountains of Xinjiang Province of China (Xu et al., 2016). In traditional Chinese medicine, *S. involucrata* has long been used to enhance blood circulation, expelling wind, eliminating heat and dampness (Chik et al., 2015), and *S. involucrata* also is used to improve stomachache, menstrual disorders and expelling wind in China (Xu et al., 2016). Acacetin is an *O*-methylated flavone compound that can be isolated from *S. involucrata, S. tridactyla* or other Asteraceae family (Li et al., 2008; Xu et al., 2016; Zhao et al., 2016). Previous studies found that acacetin has anti-tumor effects in that it can induce apoptosis and suppress cell proliferation in gastric carcinoma cells, oral squamous cell carcinoma, and prostate cancer cells (Pan et al., 2005; Kim et al., 2014, 2015). Acacetin also significantly protects rats from ischemia/reperfusion injury (Liu et al., 2016). Our lab found that acacetin ameliorates airway inflammation and eosinophil infiltration in the lungs of asthmatic mice (Huang and Liou, 2012).

In the current study, we investigated whether acacetin modulates adipogenesis and lipolysis in differentiated adipocytes and in the visceral adipose tissue of high-fat diet- (HFD) induced obese mice. We also investigated whether acacetin reduced the inflammatory response of macrophages to adipogenic differentiation medium (DM).

## Materials and Methods

### Animals and Acacetin Administration

Acacetin (≥97% purity by HPLC), can be isolated from *S. tridactyla, S. involucrata* or other Asteraceae family (Chik et al., 2015; Li et al., 2008) was purchased from Sigma-Aldrich (St. Louis, MO, United States). All experimental animal care and housing protocols were approved by the Laboratory Animal Care Committee of Chang Gung University of Science and Technology (IACUC approval number: 2013-007). Male C57BL/6 mice (4 weeks old) were purchased from the National Laboratory Animal Center in Taiwan. Mice were fed a standard chow diet or a HFD with water, and all mice were housed in temperature-controlled environment. The 4-week-old mice were randomly subdivided into 4 groups of 8 mice who were treated as follows for 16 weeks. In the N group, the mice were fed a normal diet (11.4% fat) and received DMSO by intraperitoneal injection. In the HFD group, the mice were fed an HFD diet (60% fat) and received DMSO by intraperitoneal injection. In the AC5 group, the mice were fed an HFD (60% fat) and received 5 mg/kg acacetin dissolved in DMSO by intraperitoneal injection. Finally, in the AC10 group, the mice were fed an HFD (60% fat) and received 10 mg/kg acacetin dissolved in DMSO by intraperitoneal injection. The intraperitoneal injections were performed twice a week for 10 weeks (from age 11 weeks to age 20 weeks).

### Histological Analyses of Adipocyte Tissue

For histological analysis, the mice were sacrificed, and the adipose tissue was fixed in formalin and embedded in paraffin, as described previously (Huang et al., 2017). Adipocyte tissue sections were stained with hematoxylin and eosin (HE) and were observed with an optical microscope (Olympus, Tokyo, Japan).

### Cell Culture

The 3T3-L1 murine pre-adipocyte cell line and RAW264.7 murine macrophage cell line were purchased from the Bioresource Collection and Research Center (BCRC, Taiwan).

3T3-L1 cells were cultured in DMEM (Invitrogen-Gibco^TM^, Paisley, Scotland) supplemented with 10% heat-inactivated fetal calf serum (Invitrogen-Gibco^TM^). The RAW 264.7 cells were routinely cultured in DMEM supplemented with 10% heat-inactivated fetal bovine serum (Biological Industries, Haemek, Israel). All cells were incubated at 37°C in a 5% CO2 atmosphere and subcultured twice a week.

### Cell Viability Assay

For cell culture experiments, acacetin was dissolved in DMSO, and the final DMSO concentration was ≤0.1%. Cell viability was evaluated by the MTT tetrazolium assay as described previously (Chang et al., 2012). In brief, 3T3-L1 cells were treated with various concentrations of acacetin for 24 h. The culture medium was removed, the MTT solution was added (5 mg/ml, Sigma), and the cells were incubated at 37°C for 4 h. Isopropanol was added to the culture plate and absorbance at 570 nm was detected using a spectrophotometer (Multiskan FC, Thermo Fisher Scientific, Waltham, MA, United States).

### Adipocyte Differentiation

3T3-L1 pre-adipocytes were seeded in 6-well plates and stimulated with 0.5 mM 1-isobutyl-3-methylxanthine, 1 μM dexamethasone, and 10 μg/ml insulin for 2 days. The DMEM was then replenished with the addition of 10 μg/ml insulin for 2 days, and the medium was replaced with DMEM without insulin every 2 days until day 8. On day 8, the differentiated adipocytes were treated with various concentrations of acacetin (3–100 μM) for 24 h.

### RAW 264.7 Cells Cultured with Differentiated Medium from 3T3-L1 Cells

Differentiated 3T3-L1 adipocytes were cultured with DMEM medium for 24 h, then the cultured medium (DM) was collected as described previously (Huang et al., 2013). RAW 264.7 cells were cultured in DM plus acacetin for 1 h to measure the effects of acacetin on the NF-κB and mitogen-activated protein kinase (MAPK) signaling pathways or for 24 h to evaluate the effects on COX-2 and cytokine protein levels and on NO levels.

### Determination of NO Production

RAW 264.7 cells were cultured in DM plus acacetin for 1 h to collect medium for detected NO production. The nitrite measured as an indicator of NO in culture medium by Griess reagentas previously described (Chang et al., 2012). The levels of nitrite determined by absorbance at 570 nm using a microplate reader (Multiskan FC, Thermo Fisher Scientific).

### Glycerol Production Assay

Glycerol was measured using the Glycerol Assay Kit (Sigma) according to the manufacturer’s protocol. Differentiated adipocytes were treated with various concentrations of acacetin for 24 h. Culture medium was collected and treated with the assay reagent. The levels of glycerol were determined by absorbance at 570 nm using a microplate reader (Multiskan FC, Thermo Fisher Scientific).

### Oil Red O Staining

Differentiated adipocytes were treated with acacetin for 24 h and fixed with formalin as described previously (Liou et al., 2015). The cells were stained with oil red O stain, and the oil droplets were observed using microscopy (Olympus). The cell culture plates were treated with isopropanol, and lipid accumulation was determined by absorbance at 490 nm using a microplate reader (Multiskan FC, Thermo Fisher Scientific).

### Western Blot Analysis

Cells were treated with acacetin and lysed using protein lysis buffer (Sigma). Equal amounts of protein were separated on 8–10% SDS-PAGE gels and transferred onto polyvinylidene fluoride membranes (PVDF; Millipore, Billerica, MA, United States). The PVDF membranes were blocked, incubated with primary antibodies overnight at 4°C, incubated with secondary antibodies, and the signal was detected with Luminol/Enhancer solution (Millipore). Protein bands were quantitated using the BioSpectrum 600 system (UVP, Upland, CA, United States). The primary antibodies included antibodies that recognized the following proteins: phosphorylated-AMPKα, AMPK, IκBα, phosphorylated-IκBα, p65, and lamin B1 (Santa Cruz, CA, United States); ERK1/2, phosphorylated-ERK1/2 (pERK1/2), JNK, phosphorylated-JNK (pJNK), p38, phosphorylated-p38 (pp38), fatty acid synthase (FAS), fatty acid binding protein (aP2), sirtuin 1 (Sirt1), SREBP-1c, and lipoprotein lipase (LPL) (Cell Signaling Technology, Danvers, MA, United States); acetyl CoA carboxylase-1 (ACC-1), phosphorylated-ACC-1 (pACC-1), adipose triglyceride lipase (ATGL), C/EBPα, C/EBPβ, PPAR-α, PPAR-γ, hormone-sensitive lipase (HSL), phosphorylated-HSL (pHSL) (Abcam, Cambridge, MA, United States); and β-actin (Sigma).

### RNA Isolation and Real-Time PCR to Assess Gene Expression

RNA extracted from 3T3-L1 adipocytes using TRIzol reagent (Life Technologies, Carlsbad, CA, United States), and cDNA synthesized using a cDNA synthesis kit (Life Technologies). The specific DNA was amplified using real-time PCR with the SYBR Green Master kit (Bio-Rad Laboratories, Hercules, CA, United States) and a spectrofluorometric thermal cycler (iCycler; Bio-Rad Laboratories). The specific primers that were used for real-time PCR are shown in **Table 1**.

**Table 1:** Primers used for the real-time PCR analysis of genes involved in adipogenesis and lipolysis.

Gene	Primers	5′–3′ sequence
C/EBPα	Forward	GACTTGGTGCGTCTAAGATGAG
	Reverse	TAGGCATTGGAGCGGTGAG
C/EBPβ	Forward	GTCCAAACCAACCGCACAT
	Reverse	CAGAGGGAGAAGCAGAGAGTT
FAS	Forward	ATCCTGGCTGACGAAGACTC
	Reverse	TGCTGCTGAGGTTGGAGAG
HSL	Reverse	GCTCACGGTCACCATCTCA
	Forward	CTCCTCACTGTCCTGTCCTTC
LPL	Forward	GGCTCTGCTTGAGTTGTAGAA
	Reverse	GGCATCTGAGAACGAGTCTTC
PPAR-α	Forward	GGAGCGTTGTCTGGAGGTT
	Reverse	GAAGTGGTGGCTAAGTTGTTGA
PPAR-γ	Forward	GATGACAGCGACTTGGCAAT
	Reverse	TGTAGCAGGTTGTCTTGAATGT
SREBP-1c	Forward	CTGTTGGTGCTCGTCTCCT
	Reverse	TTGCGATGCCTCCAGAAGTA
β-actin	Forward	AAGACCTCTATGCCAACACAGT
	Reverse	AGCCAGAGCAGTAATCTCCTTC

### Measurement of Proinflammatory Cytokines and Chemokines

ELISAs were performed as described previously (Huang et al., 2013). 3T3-L1 adipocytes were pretreated with acacetin for 1 h, then TNF-α (5 ng/ml) was added and the cells were cultured for 24 h. The supernatants were collected and assayed using ELISA kits specific for CCL5 and MCP-1 (R&D Systems, Minneapolis, MN, United States). In addition, RAW 264.7 cells were treated with acacetin in DM for 24 h, and the supernatant was collected to evaluate the levels of TNF-α, IL-6, and MCP-1 (R&D Systems). The levels were determined by the absorbance at 450 nm using a microplate reader microplate reader (Multiskan FC, Thermo).

### Statistical Analysis

Statistical analyses were performed using one-way ANOVA with Dunnett’s *post hoc* test. The results are reported as means ± standard deviation (SD). *P*-values less than 0.05 were considered statistically significant.

## Results

### The Viability of 3T3-L1 Cells Treated with Acacetin

The MTT assay was used to evaluate the cytotoxicity of acacetin in 3T3-L1 adipocytes.

Acacetin at concentrations ≤ 100 μM showed no significant cytotoxicity in 3T3-L1 cells (data not shown). Therefore, 3–100 μM acacetin was used in the subsequent *in vitro* experiments.

### The Effect of Acacetin on Lipid Accumulation in Adipocytes

**FIGURE 1:**
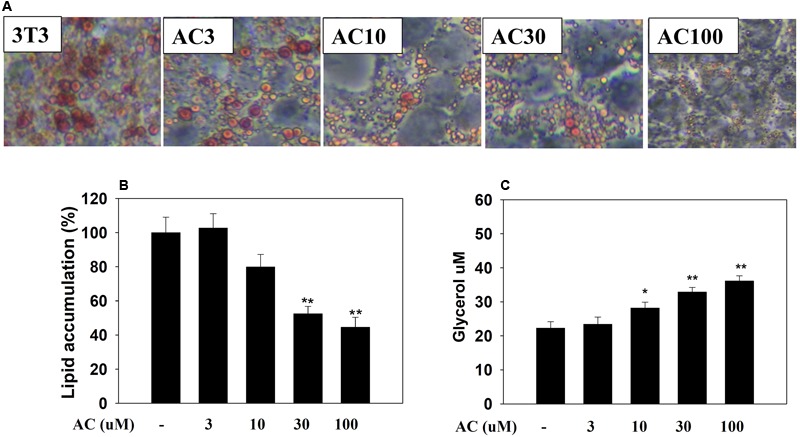
Acacetin (AC) (3–100 μM) reduces adipogenesis in 3T3-L1 cells. **(A)** Oil red O stain was used to evaluate lipid accumulation in cells treated with the indicated concentration of acacetin. **(B)** 3T3-L1 cells were treated with isopropanol and lipid accumulation was measured using the absorbance at OD 490 nm. Lipid accumulation in control group cells compared to acacetin-treated 3T3-L1 cells. **(C)** Glycerol concentrations were determined in the culture medium. Data are presented as means ± SD, *n* = 6. ^∗^*P* < 0.05, ^∗∗^*P* < 0.01 compared with the control group.

3T3-L1 adipocytes were treated with 0, 3, 10, 30, or 100 μM acacetin (termed AC3, AC10, AC30, and AC100), and lipid accumulation was assessed by oil red O staining. Microscopy observations showed that acacetin treatment reduced lipid droplets in differentiated 3T3-L1 cells (**Figure 1A**). Lipid accumulation in adipocytes was measured by treating the cells with isopropanol to release the oil droplets into the solution. Acacetin significantly reduced lipid accumulation compared to control cells (**Figure 1B**). Triglycerides in adipocytes are broken down to produce free fatty acid and glycerol (Rajan et al., 2014). We found that acacetin significantly increased the glycerol levels in the culture medium (AC3: 23.4 ± 2.2 μM, *P* = 0.65; AC10: 28.2 ± 2.7 μM, *P* < 0.05; AC30: 32.9 ± 1.4 μM, *P* < 0.01; AC100: 36.1 ± 3.1 μM, *P* < 0.01 *vs.* control: 22.3 ± 1.8 μM, respectively) (**Figure 1C**).

### The Effect of Acacetin on the Expression of Adipogenesis-Related Transcription Factor Genes and Proteins in Differentiated Adipocytes

**FIGURE 2:**
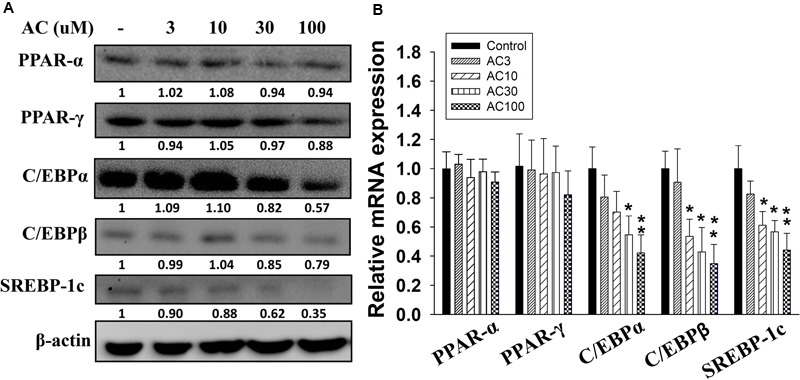
The effect of acacetin (AC) (3–100 μM) on the expression of transcription factors involved in adipogenesis in 3T3-L1 cells. **(A)** Differentiated 3T3-L1 cells were treated with acacetin for 24 h. The protein expression of PPAR-α, PPAR-γ, C/EBPα, C/EBPβ, and SREBP-1c was determined using Western blot analysis (*n* = 3). β-actin was used as an internal control. **(B)** The gene expression of the transcription factors was measured using real-time PCR. β-actin was used as an internal control. Data are presented as means ± SD. ^∗^*P* < 0.05, ^∗∗^*P* < 0.01 compared to differentiated 3T3-L1 cells (control group).

Western blot analysis showed that differentiated adipocytes treated with acacetin did not express significantly lower levels of the PPAR-α and PPAR-γ proteins than the control cells (**Figure 2A**). However, acacetin significantly decreased the protein expression of C/EBPα, C/EBPβ, and SREBP-1c. Real-time PCR analysis showed that differentiated adipocytes treated with acacetin had lower mRNA levels of C/EBPα, C/EBPβ, and SREBP-1c but not lower mRNA levels of PPAR-α and PPAR-γ than the control cells (**Figure 2B**).

### The Effect of Acacetin on the Expression of Adipogenesis- and Lipolysis-Associated Genes and Proteins in 3T3-L1 Cells

**FIGURE 3:**
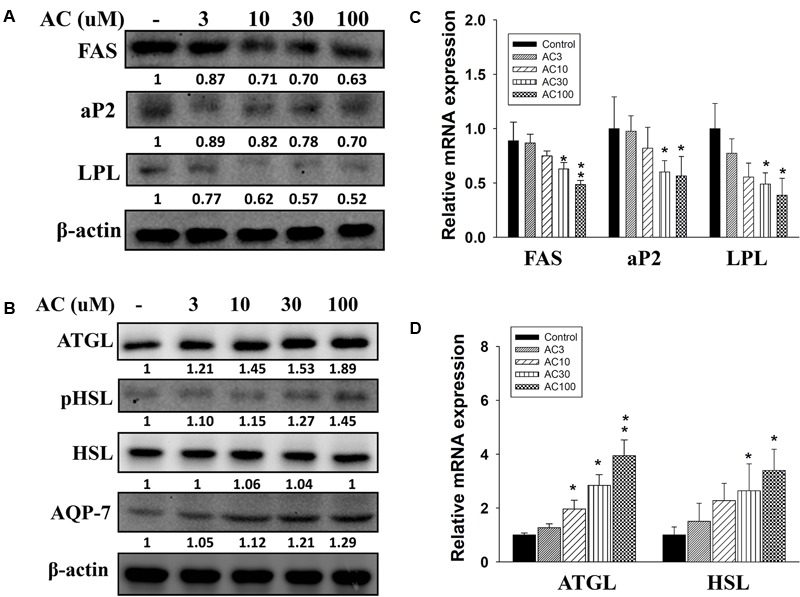
The effect of acacetin (AC) (3–100 μM) on the expression of proteins involved in adipogenesis and lipolysis in differentiated 3T3-L1 adipocytes. **(A)** The protein expression of FAS, LPL, and aP2 and the protein expression of **(B)** ATGL, phosphorylated HSL (pHSL), and AQP7 was determined using Western blot analysis (*n* = 3 per group). β-actin was used as an internal control. **(C)** The gene expression of FAS, LPL, and aP2 and **(D)** the gene expression of ATGL and HSL was measured using real-time PCR. β-actin was used as an internal control. Data are presented as means ± SD. ^∗^*P* < 0.05, ^∗∗^*P* < 0.01 compared to differentiated 3T3-L1 cells (control group).

Acacetin suppressed FAS, LPL, and aP2 protein and gene expression in 3T3-L1 cells in a concentration-dependent manner (**Figures 3A,C**). Acacetin also significantly increased ATGL and pHSL protein expression and enhanced the expression of the ATGL and HSL genes compared to control cells (**Figures 3B,D**). Aquaporin-7 (AQP7) is a channel protein on the adipocyte plasma membrane that helps regulate the release of glycerol from adipocytes into serum (Lebeck, 2014). Acacetin increased AQP7 expression compared to control cells (**Figure 3B**).

### The Effect of Acacetin on the Sirt1 and AMPK Pathways

**FIGURE 4:**
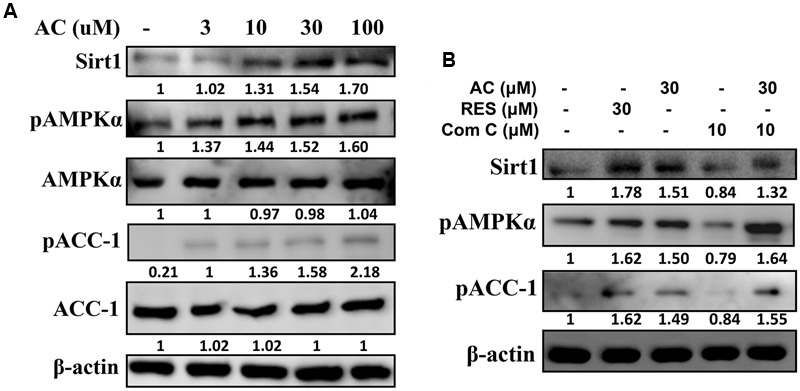
The effect of acacetin (AC) (3–100 μM) on the AMPK pathway in 3T3-L1 cells. **(A)** Differentiated 3T3-L1 adipocytes were treated with acacetin for 24 h, and the protein expression of Sirt1, phosphorylated AMPKα (pAMPKα), AMPKα, phosphorylated ACC-1 (pACC), and ACC-1 was determined using Western blot analysis (*n* = 3). AMPKα or ACC-1 was used as an internal control. **(B)** Cells were cultured with acacetin plus the AMPK inhibitor compound C (Com C), and AMPK phosphorylation and Sirt1 and ATGL protein expression were assessed. Resveratrol (RES) was used as the sirt1 positive control. β-actin was used as an internal control.

Compared to control cells, acacetin significantly increased Sirt1 expression in 3T3-L1 cells and also increased the phosphorylation of AMPKα and ACC-1 in a concentration-dependent manner (**Figure 4A**). The effects of acacetin on 3T3-L1 cells were similar to the effects of resveratrol (an AMPK activator) in that both agents enhanced Sirt1 expression and both increased the pAMPKα and pACC-1 levels (**Figure 4B**). Furthermore, the levels of pAMPK, pACC-1, and Sirt1 were restored in 3T3-L1 cells co-treated with compound C (an AMPK inhibitor) and acacetin compared to the levels in cells treated only with compound C (**Figure 4B**).

### The Effect of Acacetin on Chemokine Production in TNF-α-Induced 3T3-L1 Cells

**FIGURE 5:**
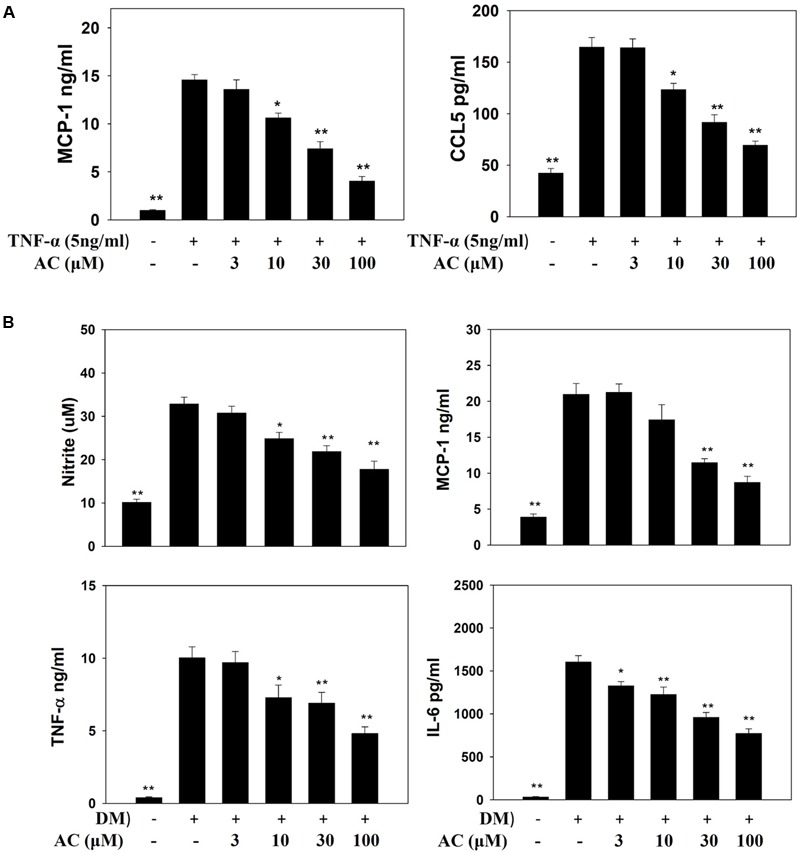
The effect of acacetin (AC) (3–100 μM) on nitrite, chemokines, and cytokine levels as measured by ELISA. **(A)** The protein expression of MCP-1 and CCL5 was measured in TNF-α-induced 3T3-L1 cells treated with acacetin. Data are presented as means ± SD. ^∗^*P* < 0.05, ^∗∗^*P* < 0.01 compared to differentiated 3T3-L1 cells (control group). **(B)** Macrophages were cultured in differentiated media (DM) and treated with acacetin for 24 h, then the levels of nitrite, MCP-1, IL-6, and TNF-α were determined. Data are presented as means ± SD (*n* = 6); ^∗^*P* < 0.05, ^∗∗^*P* < 0.01 compared with the ‘macrophages cultured in DM’ group.

Previous studies showed that TNF-α induces 3T3-L1 cells to become insulin-resistant adipocytes that release the chemokines MCP-1 and CCL5 and increase macrophage infiltration into adipocyte tissue (Smith and Minson, 2012; Anusree et al., 2015). Here we found that TNF-α stimulated 3T3-L1 adipocytes to release MCP-1 and CCL5. Acacetin significantly reduced the levels of CCL5 and MCP-1 compared to TNF-α-induced 3T3-L1 adipocytes (**Figure 5A**).

### Acacetin Reduces the Levels of Inflammatory Mediators in RAW 264.7 Cells Cultured with DM

**FIGURE 6:**
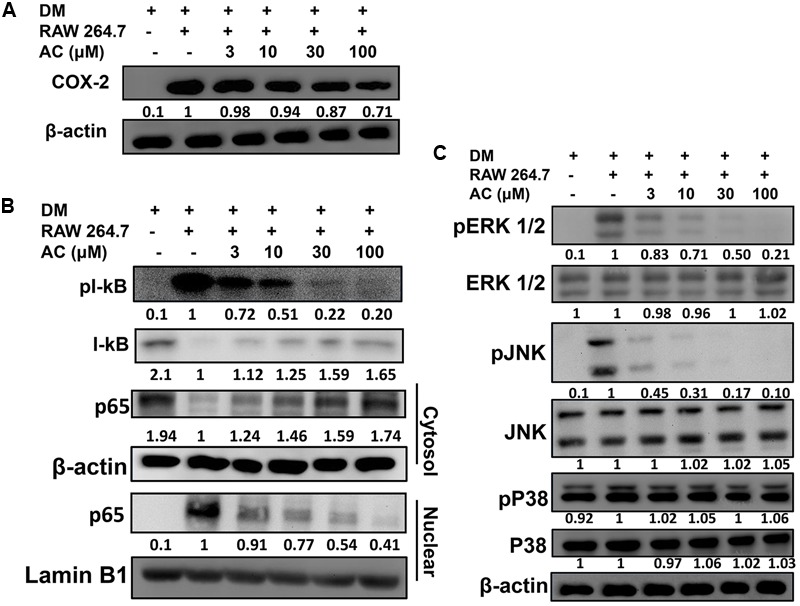
The effect of acacetin (AC) (3–100 μM) on **(A)** COX-2 protein expression in RAW 264.7 cells cultured with DM for 24 h. **(B)** Acacetin inhibited the phosphorylation of IκB-α and the nuclear translocation of NF-κB in RAW264.7 cells. **(C)** Acacetin inhibited the phosphorylation of MAPK in RAW264.7 cells. For all experiments, *n* = 3. β-actin was used as internal control in cytosol, and Lamin B1 was used as internal control in nuclear.

Next we investigated whether acacetin could suppress the inflammatory response of macrophages to the fatty acids or adipokines released by adipocytes. RAW 264.7 macrophages were treated with acacetin or left untreated and then cultured in DM from differentiated 3T3-L1 cells for 24 h. We found that the RAW 264.7 cells cultured in DM medium showed significantly increased levels of nitrite, TNF-α, IL-6 and MCP-1, but acacetin treatment significantly reduced the levels of these inflammatory mediators (**Figure 5B**). Acacetin also decreased COX-2 expression compared to the DM only group (**Figure 6A**).

### The Effect of Acacetin on the NF-κB and MAPK Signaling Pathways in RAW264.7 Macrophages Cultured in DM

RAW 264.7 macrophage cells were treated with acacetin or left untreated and then cultured in DM from differentiated 3T3-L1 cells for 24 h. Acacetin reduced the levels of pIκB-α and attenuated the transport of the NF-κB subunit p65 into the nucleus (**Figure 6B**). Acacetin also significantly inhibited the phosphorylation of ERK1/2 and JNK but did not reduce p38 phosphorylation (**Figure 6C**).

### Acacetin Decreased the Weight of Visceral Adipocyte Tissue and the Body Weight of HFD-Induced Obese Mice

**FIGURE 7:**
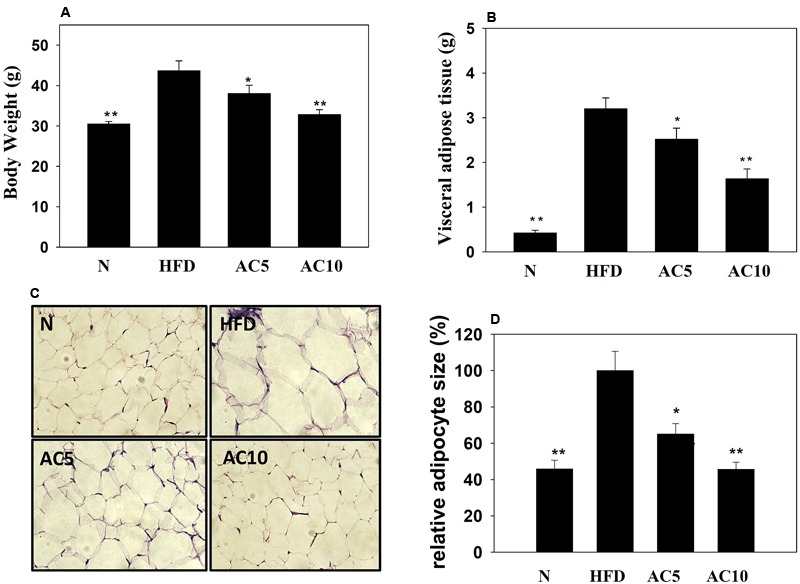
Acacetin (AC) (5 or 10 mg/kg) decreased the body weight **(A)** and the visceral adipocyte tissue weight **(B)** of HFD-induced obese mice. **(C)** Histological sections of visceral adipocyte tissue in mice. **(D)** Adipocyte size in control HFD-induced mice compared to acacetin-treated HFD-induced obese mice. Data are presented as means ± SD, *n* = 8. ^∗^*P* < 0.05, ^∗∗^*P* < 0.01 compared with HFD-induced obese mice.

We investigated the effects of acacetin on HFD-induced obese mice and found that acacetin treatment significantly reduced the body weight and visceral adipocyte tissue weight of obese mice (**Figures 7A,B**). Histological analysis of the adipocyte tissue showed that acacetin decreased lipid accumulation and adipocyte size compared to untreated HFD-induced obese mice (**Figures 7C,D**).

### The Effect of Acacetin on Regulated Gene Expression in Visceral Adipocyte Tissue

**FIGURE 8:**
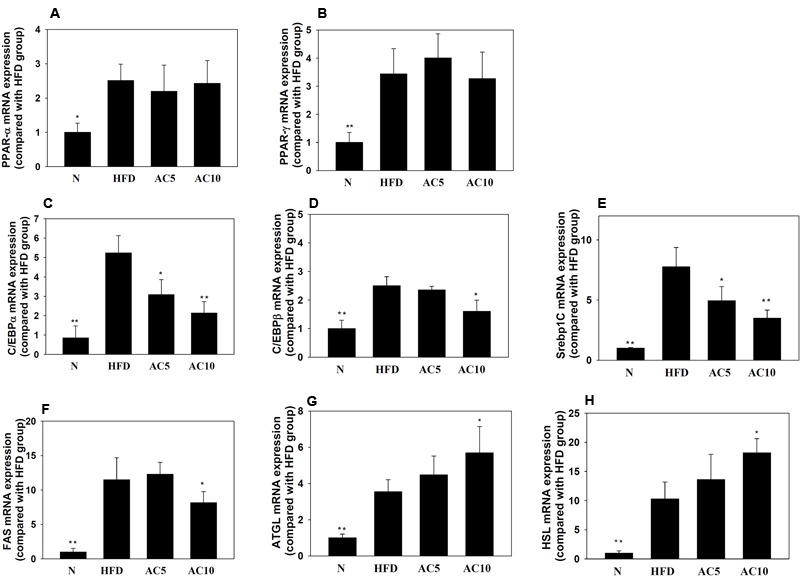
The effect of acacetin (AC) (5 or 10 mg/kg) on gene expression in the visceral adipocyte tissue of mice. Real-time PCR analysis was used to determine the gene expression of **(A)** PPARα, **(B)** PPARγ, **(C)** CEBP/α, **(D)** C/EBPβ, **(E)** SREBP-1c, **(F)** FAS, **(G)** ATGL, and **(H)** HSL in murine visceral adipocyte tissue. Data are presented as means ± SD, *n* = 8. ^∗^*P* < 0.05, ^∗∗^*P* < 0.01 compared with HFD-induced obese mice.

Real-time PCR analysis showed that acacetin did not significantly increase or decrease PPARα and PPARγ gene expression in mouse visceral adipocyte tissue compared with the HFD group. Interestingly, acacetin significantly decreased the gene expression of C/EBPα, C/EBPβ, SREBP-1c, and FAS, and it also significantly increased the gene expression of ATGL and HSL compared with the HFD group (**Figure 8**).

## Discussion

Obesity is an important causal factor in many chronic diseases, including cardiovascular diseases, hypertension, hyperlipidemia, diabetes, and cancer (Singer and Lumeng, 2017) (Morrison and Kleemann, 2015; Geeraerts et al., 2017). Overnutrition can cause energy hoarding, resulting in the accumulation of energy stores such as glycogen or triglycerides in liver, muscle, and adipose tissue. Importantly, although adipose tissue accumulates lipids, excessive lipid accumulation in adipose tissue interferes with the normal physiological function of adipocytes and increases the secretion of adipokines to levels that can contribute to the development of many chronic diseases (Forte et al., 2012; Singer and Lumeng, 2017).

Many studies found that some natural products may improve cardiovascular diseases and diabetes in obesity. Curcumin could modulate hypolipidemic effects, inhibit liver inflammation and decrease lipid accumulation of liver and adipose tissue in HFD-induced obese mice (Zingg et al., 2013). Resveratrol also was confirmed that might be a better polyphenol to prevent cardiovascular disease in obese individuals (Huang et al., 2016).

Quercetin could regulate glucose uptake in skeletal muscle cell and hepatocytes for improved type 2 diabetes (Wang et al., 2013; Dhanya et al., 2017). In the present study, we investigated whether acacetin has anti-obesity effects in 3T3-L1 adipocytes and in HFD-induced obese mice. In this preliminary study, we found that acacetin significantly decreased the body weight and adipocyte tissue weight of HFD-induced mice. Acacetin significantly reduced lipid accumulation and attenuated the expression of the adipogenesis-related transcription factors C/EBP and SREBP-1c. It also downregulated the expression of lipogenesis enzymes, increased the expression of lipolysis enzymes, and enhanced the phosphorylation of AMPKα and ACC-1 in adipocytes. These results suggest that acacetin significantly reduces adipogenesis in adipocytes and has an anti-obesity effect in obese mice.

Adipogenesis was the process that the differentiation of preadipocytes changed into mature adipocytes. Previous studies found that 3T3-L1 preadipocytes require the sequential action of adipogenesis-related transcription factors to induce and regulate their differentiation into mature adipocytes (Guo et al., 2015; van der Krieken et al., 2015). PPARs (PPARα, PPARβ, and PPARγ) are important transcription factors that regulate lipogenesis and affect adipocyte differentiation (Janani and Ranjitha Kumari, 2015). PPARα and PPARβ regulate the early stages of adipocyte differentiation, while PPARγ may be involved in later stages (Tang and Lane, 2012; Rajan et al., 2014). However, the present study found that acacetin did not suppress PPARα and PPARγ protein and gene expression in mature adipocytes. In addition, acacetin did not reduce the gene expression of PPARα and PPARγ in adipocyte tissue in obese mice. In recent years, many studies have suggested that PPARα activation is involved in lipid metabolism in adipose and liver tissue and that it increases the β-oxidation of fatty acids for lipid catabolism (Barbosa-da-Silva et al., 2015). PPARγ is also thought to improve carbohydrate and lipid metabolism and thereby block the development of insulin resistance in diabetic or obese mice (Zhou et al., 2016). Here we found that acacetin did not regulate fatty acid synthesis or adipocyte differentiation via PPARα or PPARγ.

Hence, we also investigated the possible involvement of other transcription factors in adipocyte differentiation. C/EBP and SREBP-1c are two transcription factors that are involved in lipogenesis in the adipocyte differentiation process (Mota de Sa et al., 2017).

Early stage of adipocyte differentiation, dexamethasone stimulated C/EBPβ expression for maintained the early stage of gene expression (Ali et al., 2013). Next, C/EBPα and SREBP-1c would control and increase lipid accumulation (Mota de Sa et al., 2017).

Acacetin significantly decreased C/EBPα, C/EBPβ, and SREBP-1c expression in mature adipocytes *in vitro* and in the adipocyte tissue of obese mice *in vivo*. Our results thus suggested that acacetin attenuated lipogenesis and had an anti-obesity effect in obese mice mainly by blocking the action of C/EBPs and SREBP-1c. Notably, SREBP-1c and C/EBPs bind to the promoter of FAS to promote lipogenesis and to increase aP2 and LPL expression in adipocytes (Li G. et al., 2017; Mota de Sa et al., 2017). aP2 is expressed in mature adipose tissue where it regulates lipid and glucose metabolism (Wang et al., 2015). FAS is a multifunctional enzyme in lipogenesis that catalyzes and synthesizes long-chain fatty acids from acetyl-CoA (Moseti et al., 2016). LPL hydrolyzes lipoproteins from chylomicrons and very low-density lipoproteins to create more free triglycerides in serum; notably, high triglyceride levels are implicated in the development of cardiovascular disease (Mota de Sa et al., 2017). Acacetin reduces aP2, FAS, and LPL, which decreases the transport of fatty acids and lipid synthesis. An earlier study found that acacetin improves cardiovascular disease and protects against ischemia/reperfusion injury (Liu et al., 2016). We speculate that acacetin reduces LPL and, in doing so, may prevent high serum levels of triglycerides and have a protective effect on the cardiovascular system.

Reducing lipid accumulation and increasing lipid catabolism in adipocyte tissue has an anti-obesity effect in obese mice (Ali et al., 2013). In the present study, acacetin decreased adipocyte tissue weight and adipocyte size in HFD-induced obese mice and reduced droplet accumulation in mature 3T3-L1 adipocytes. Acacetin increased AQP-7 expression, which exacerbated lipolysis in adipocytes and increased the release of glycerol into the culture medium. Acacetin significantly increased the protein expression of ATGL and the phosphorylation of HSL in mature adipocytes. Acacetin also increased the gene expression of ATGL and HSL in the adipocyte tissue of HFD-induced mice and in mature 3T3-L1 adipocytes. Therefore, acacetin has the potential to break down triglycerides and reduce the weight of obese mice.

AMPK is an energy sensor that is involved in the regulation of lipid and glucose metabolism (Lim et al., 2010), while Sirt1 coordinates the effects of the PGC1-α/ERR-α complex in modulating adipogenesis (Cai et al., 2016). Resveratrol is a Sirt1 inducer that activates the AMPK pathway in adipocytes in obese mice and in 3T3-L1 cells (Chen et al., 2012). In adipocytes and hepatocytes, AMPK suppresses fatty acid synthesis and enhances HSL phosphorylation to promote lipolysis (Gaidhu et al., 2009). Our experimental results demonstrated that acacetin significantly increased the phosphorylation of AMPK and Sirt1 and increased ACC-1 phosphorylation. This blocked FAS expression, which suppressed fatty acid synthesis. In 3T3-L1 adipocytes treated with compound C, acacetin restored ACC-1 and AMPK phosphorylation levels and Sirt1 expression levels. Therefore, acacetin may promote AMPK activation and block lipogenesis in adipocytes.

The present study found that acacetin could break down triglycerides into glycerol and free fatty acids, and the free fatty acids were released into the culture medium. A previous study showed that mature adipocytes release fatty acids or adipokines to induce an inflammatory response in macrophages via toll-like receptor 4 and via activated MAPK and NF-κB signaling pathways (Bao et al., 2015). Adipokines also attract more macrophages, which migrate toward and infiltrate into adipocytes (Ouchi et al., 2011). This prompted us to investigate whether acacetin attenuated the inflammatory response of macrophages by blocking the production or release of adipokines or free fatty acids. Interestingly, acacetin significantly reduced the production of inflammatory mediators, including NO, COX-2, IL-6, TNF-α, and MCP-1, and acacetin also inhibited the NF-κB and MAPK inflammatory signaling pathways in DM-stimulated macrophages. A previous study demonstrated that free fatty acids induced JNK phosphorylation in macrophages (Nguyen et al., 2007). Our results demonstrated that acacetin reduced the phosphorylation of IκB-β, pERK, and pJNK. However, acacetin did not attenuate p38 phosphorylation in DM-stimulated macrophages. Hence, acacetin could inhibit the inflammatory response by blocking JNK, ERK, and NF-κB expression in DM-stimulated macrophages.

Excessive lipid accumulation induces insulin resistance in adipocytes, hepatocytes, and skeletal muscle cells (Zhou et al., 2016). Insulin-resistant adipocytes secrete CCL5 and MCP1, which attract macrophages that infiltrate into adipose tissue and which induce macrophage activation and inflammation (Olefsky and Glass, 2010). A previous study found that inflammatory macrophages release TNF-α, inducing insulin resistance in adipocytes, which then secrete more CCL5 and MCP-1 and attract more macrophages that infiltrate into adipose tissue (Huang et al., 2013). The result is a vicious cycle involving macrophages and adipocytes that can lead to more serious chronic inflammatory diseases and to insulin resistance in obesity. Here we found that mature 3T3-L1 adipocytes that were stimulated with TNF-α released significantly more CCL5 and MCP-1 than mature 3T3-L1 adipocytes. These findings provide evidences that acacetin suppressed the levels of CCL5 and MCP-1, which could attenuate or improve insulin resistance in adipocytes.

## Conclusion

Acacetin reduced both the body weight and visceral adipose tissue weight of HFD-induced obese mice and suppressed the expression of genes involved in lipogenesis in murine visceral adipose tissue. *In vitro*, acacetin suppressed the expression of transcription factors involved in lipid synthesis in mature 3T3-L1 adipocytes and increased lipolysis by increasing ATGL and HSL expression. Moreover, acacetin reduced macrophage inflammation when macrophages were cultured with DM from mature 3T3-L1 adipocytes. These findings provide evidences that acacetin inhibit adipogenesis in adipocytes and in obese mice, and acacetin is a natural product that has potential anti-obesity effects.

## Author Contributions

Designed the experiments: C-JL, S-JW, and W-CH; Performed the experiments: C-JL, S-JW, and C-YC; Analysis and interpretation of data: L-CC and K-WY; Drafting the manuscript: C-JL, L-CC, and W-CH.

## Conflict of Interest Statement

The authors declare that the research was conducted in the absence of any commercial or financial relationships that could be construed as a potential conflict of interest.
